# Diagnostic value of the novel CMR parameter “myocardial transit-time” (MyoTT) for the assessment of microvascular changes in cardiac amyloidosis and hypertrophic cardiomyopathy

**DOI:** 10.1007/s00392-020-01661-6

**Published:** 2020-05-05

**Authors:** Grigorios Chatzantonis, Michael Bietenbeck, Anca Florian, Claudia Meier, Philipp Stalling, Dennis Korthals, Holger Reinecke, Ali Yilmaz

**Affiliations:** Department of Cardiology I, University Hospital Münster, Albert-Schweitzer-Campus 1, Building A1, 48149 Münster, Germany

**Keywords:** MVD, CMD, CMR, HCM, ECV, MyoTT

## Abstract

**Background:**

Coronary microvascular dysfunction (CMD) is present in various non-ischemic cardiomyopathies and in particular in those with left-ventricular hypertrophy. This study evaluated the diagnostic value of the novel cardiovascular magnetic resonance (CMR) parameter “myocardial transit-time” (MyoTT) in distinguishing cardiac amyloidosis from other hypertrophic cardiomyopathies.

**Methods:**

*N =* 20 patients with biopsy-proven cardiac amyloidosis (CA), *N =* 20 patients with known hypertrophic cardiomyopathy (HCM), and *N =* 20 control patients without relevant cardiac disease underwent dedicated CMR studies on a 1.5-T MR scanner. The CMR protocol comprised cine and late-gadolinium-enhancement (LGE) imaging as well as first-pass perfusion acquisitions at rest for MyoTT measurement. MyoTT was defined as the blood circulation time from the orifice of the coronary arteries to the pooling in the coronary sinus (CS) reflecting the transit-time of gadolinium in the myocardial microvasculature.

**Results:**

MyoTT was significantly prolonged in patients with CA compared to both groups: 14.8 ± 4.1 s in CA vs. 12.2 ± 2.5 s in HCM (*p =* 0.043) vs. 7.2 ± 2.6 s in controls (*p* < 0.001). Native T1 and extracellular volume (ECV) were significantly higher in CA compared to HCM and controls (*p* < 0.001). Both parameters were associated with a higher diagnostic accuracy in predicting the presence of CA compared to MyoTT: area under the curve (AUC) for native T1 = 0.93 (95% confidence interval (CI) = 0.83–1.00; *p* < 0.001) and AUC for ECV = 0.95 (95% CI = 0.88–1.00; *p* < 0.001)—compared to the AUC for MyoTT = 0.76 (95% CI = 0.60–0.92; *p =* 0.008). In contrast, MyoTT performed better than all other CMR parameters in differentiating HCM from controls (AUC for MyoTT = 0.93; 95% CI = 0.81–1.00; *p =* 0.003 vs. AUC for native T1 = 0.69; 95% CI = 0.44–0.93; *p =* 0.20 vs. AUC for ECV = 0.85; 95% CI = 0.66–1.00; *p =* 0.017).

**Conclusion:**

The relative severity of CMD (measured by MyoTT) in relationship to extracellular changes (measured by native T1 and/or ECV) is more pronounced in HCM compared to CA—in spite of a higher absolute MyoTT value in CA patients. Hence, MyoTT may improve our understanding of the interplay between extracellular/intracellular and intravasal changes that occur in the myocardium during the disease course of different cardiomyopathies.

## Introduction

Systemic amyloidoses comprise a growing group of disorders caused by the extracellular deposition of misfolded proteins in various organs such as heart, liver, kidney, skin, eyes, lungs, and nervous system, thereby resulting in different clinical manifestations [1]. Cardiac involvement varies among types of amyloidosis, most commonly found in immunoglobulin light chain (AL) and transthyretin (ATTR) amyloidosis [2]. Infiltration of the human heart by amyloid deposits is associated with a high morbidity and poor prognosis [3, 4]. Therefore, diagnosis of cardiac involvement at the early stages and initiation of a targeted therapy (if possible) may tremendously affect individual prognosis.

Cardiac amyloidosis (CA) is characterized by rapid progressive heart failure, arrhythmias, orthostatic dysregulation, and conduction abnormalities [5]. Due to the accumulation of amyloid in the myocardial interstitium, relaxation and compliance of the heart muscle are impaired, resulting in a restrictive form of cardiomyopathy [6, 7] that is also called “stiff heart” syndrome [8]. Noteworthy, amyloid fibrils cannot only deposit in the atrial and ventricular walls, in the valves and the conduction system, but also accumulate in the vessel walls of the coronary and microvascular system [9]. In this context, infiltration and accumulation of amyloid in coronary vessel walls may result in impaired vasodilation, microinfarction, luminal obliteration, and eventually reduced myocardial perfusion [10].

In contrast to CA, other forms of hypertrophic cardiomyopathy (HCM) are more frequent [11]: In particular, “conventional” HCM that is mostly caused by mutations in genes encoding proteins of the cardiac sarcomere is an important differential diagnosis in patients with hypertrophied left ventricles not caused by arterial hypertension [12]. Patients with such “conventional” HCM forms can be either asymptomatic or show symptoms of heart failure (HF), chest pain, or arrhythmias [13]. Histologically, “conventional” HCM forms are characterized by myocardial fibrosis, disarray, and small vessel disease [14]. Noteworthy, cellular architecture is disorganized [15], and different patterns of fibrosis may occur including perivascular fibrosis and microscopic replacement scars as a result of silent microvascular ischemia leading to cell death [16, 17].

Cardiovascular magnetic resonance (CMR) plays an important role in the diagnosis and differentiation of both pathological entities (CA and HCM) that are characterized by hypertrophied ventricular walls. In case of CA, late gadolinium enhancement (LGE) imaging reveals a characteristic pattern of diffuse LGE starting from the subendocardial layer of the myocardium and eventually involving all myocardial layers and segments [18–22]. In contrast, the “conventional” HCM is characterized by a focally accentuated, rather patchy pattern of LGE in the most hypertrophied segments of the left-ventricular myocardium [23–27].

In a recent study, we introduced a novel CMR parameter called “myocardial transit-time” (MyoTT) allowing a non-invasive and very quick assessment of coronary microvascular dysfunction (CMD) [28]. Based on the promising results of this previous study, we hypothesized that MyoTT could accurately measure the changes in the myocardial microvasculature and help to differentiate predominant intra-/perivascular changes from non-vascular interstitial processes that occur in the course of the aforementioned cardiomyopathies (CA and HCM). Therefore, the diagnostic yield of the novel CMR parameter MyoTT was assessed (in comparison to the other established CMR parameters) in both patients with CA and “conventional” HCM.

## Methods

### Study population

All patients included in this prospective, single-center study who underwent a routine CMR examination for work-up of suspected non-ischemic cardiomyopathy. The first study group (CA group) comprised *N =* 20 patients with biopsy-proven cardiac amyloidosis (including both AL and ATTR subtypes). The second study group (HCM group) comprised *N =* 20 patients with “conventional” HCM showing normal LV ejection fraction (LV-EF) ≥ 50%, LV wall thickness ≥ 15 mm (that could not be explained by abnormal loading conditions), absence of LV outflow tract obstruction and of known CAD, or any other infiltrative cardiomyopathy. Patients with any history of relevant valvular disease (at least grade II in echocardiography and/or at least moderate in CMR), prosthetic valves, and congenital heart disease were excluded. In addition, a control group (*N =* 20) without any structural and functional cardiac abnormalities and a low pre-test probability of CAD was recruited. The local ethics committee approved the study protocol and written informed consent was obtained from every patient prior to the CMR study.

### CMR acquisition

**Fig. 1:**
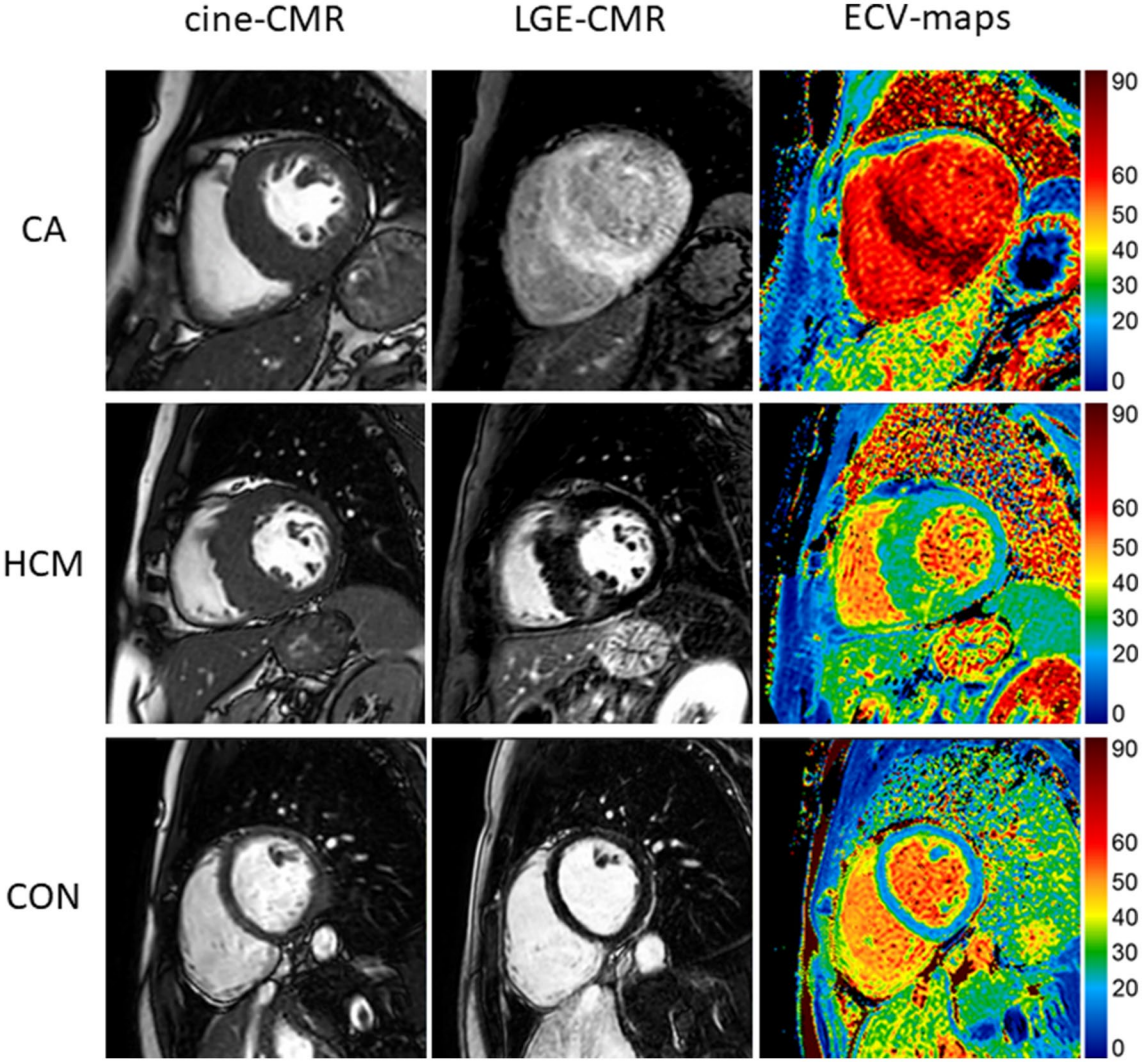
Cine imaging (first column), late gadolinium enhancement (LGE, second column), and extracellular volume fraction (ECV) maps (third column) in short-axis views of cardiac amyloidosis (CA) patients (first row), hypertrophic cardiomyopathy (HCM) patients (second row), and healthy controls (third row)

CMR imaging was performed on a 1.5-T system (Ingenia, Philips Healthcare, Best, The Netherlands) during breath-hold and with ECG-triggering. The CMR protocol (Fig. 1) included standard 2D sequences for cine imaging, myocardial resting perfusion and LGE-imaging. In addition, a modified Look Locker inversion recovery (MOLLI) T1-mapping sequence was applied in three short-axis views prior to contrast agent administration and ~ 20 min thereafter to determine extracellular volume fraction (ECV). For the measurement of MyoTT, at least one perfusion slice was planned to cover the coronary sinus (CS) and the aortic root [using 0.075 mmol/kg BW gadolinium (Gadobutrol) and a 30 ml saline flush at 4 ml/s], described in more detail elsewhere [28].

### CMR data analysis

Image analysis and interpretation were performed using commercially available software (cvi42, Circle Cardiovascular Imaging, Calgary, Alberta, Canada). Ventricular volumes and LV mass were determined by contouring short-axis cine images. For the assessment of MyoTT, the temporal difference between the arrivals of contrast agent in the aortic root and the coronary sinus was measured as described in more detail in a previous publication [28]. LGE images were visually assessed as described in more detail elsewhere [29]. For the assessment of global LV deformation, 3D LV global longitudinal strain (LV-GLS) was calculated on standard cine images. Endo- and epicardial contours were drawn on long-axis and short-axis images. All analyses were performed offline by two experienced readers blinded to each other.

### Statistical analysis

Statistical analysis was performed with SPSS (version 25.0, IBM Corp., Armonk, NY). Continuous variables are expressed as mean with ± standard deviation, whereas skewed variables as median ± interquartile range. Categorical variables are expressed as frequency with percentage. One-way ANOVA was used for comparison of normally distributed, homogenous data, and when the data failed the assumption on homogeneity of variances (Levene’s test), Welch–ANOVA was used instead. Kruskal–Wallis test was used for comparison of non-normally distributed variables. The Chi-square test with Bonferroni correction was used to compare non-continuous variables. For the assessment of the relationship between different CMR parameters, Spearman correlation was performed. Receiver-operating characteristic curves (ROC) were analyzed to assess the specificity and sensitivity of different CMR measurements to identify patients with CA within the whole cohort as well as to differentiate HCM patients from controls. A *p* value ≤ 0.05 was considered statistically significant.

## Results

### Study population

The study population characteristics are summarized in Table 1. Males and females were equally distributed in the CA and HCM group. Median age differed significantly between the CA and both the HCM and control group (70 ± 12 years in CA vs. 49 ± 18 years in HCM vs. 50 ± 16 years in controls; *p* < 0.001) as expected due to the higher prevalence of CA in elderly patients. There were no significant differences regarding major cardiovascular disease risk factors that could theoretically influence the extent of coronary microvascular dysfunction (CMD) in our patients.

**Table 1:** Patient characteristics

	CA patients	HNCM patients	Control group	*p* value (CA vs. HNCM)	*p* value (CA vs. control)
	*N =* 20	*N =* 20	*N =* 20		
Male, *N* (%)	15 (75)	16 (80)	9 (45)	1.00	0.11
Age, years	70 (± 12)	49 (± 18)	50 (± 16)	** < 0.001**	** < 0.001**
Hypertension, *N* (%)	13 (65)	7 (35)	6 (30)	0.11	0.06
Diabetes, *N* (%)	3 (15)	3 (15)	0 (0)	1.00	0.23
High cholesterol, *N* (%)	7 (35)	4 (20)	5 (25)	0.48	0.73
Current smoker, *N* (%)	1 (5)	7 (35)	4 (20)	0.09	0.34

Bold indicates *p* < 0.05

### Conventional CMR findings

All anatomic, functional, and structural CMR findings are listed in Table 2. Compared to HCM patients and controls, left-ventricular ejection fraction (LV-EF) was slightly lower in CA patients—but still within quite normal range. Cardiac index (CI) did not differ significantly between the groups. Left-ventricular hypertrophy (LVH) was present both in CA and HCM patients—with a predominantly concentric pattern of LVH in CA patients vs. an asymmetric, septally pronounced pattern of LVH in HCM patients. Moreover, a non-ischemic, diffuse subendocardial-to-transmural pattern of LGE predominantly present in the LV basal-to-midventricular segments was detected in CA patients—with a much broader myocardial extent in comparison to the focally accentuated, patchy pattern of LGE in HCM patients (47 ± 34% vs. 15 ± 11%, *p =* 0.002). No LGE was present in the control group (*p* < 0.001).

**Table 2:** Conventional CMR parameters

	CA patients	HNCM patients	Control group	*p* value (CA vs. HNCM)	*p* value (CA vs. control)
	*N =* 20	*N =* 20	*N =* 20		
LV-EF, %	53 (47–63)	64 (57–70)	60 (58–67)	**0.003**	**0.005**
LV-EDV index, ml/m^2^	81 (± 18)	72 (± 15)	75 (± 14)	0.17	0.62
LV-ESV index, ml/m^2^	37 (± 9)	27 (± 9)	28 (± 8)	**0.001**	**0.005**
LV mass index, g/m^2^	91 (80–114)	79 (62–99)	50 (45–56)	0.55	** < 0.001**
Max. LV wall thickness, mm	19 (16–21)	17 (15–26)	9 (8–11)	1.00	** < 0.001**
Cardiac Index, (l/min)/m^2^	3.1 (± 0.6)	3.4 (± 0.5)	3.6 (± 0.8)	0.96	0.11
RV-EDV index, ml/m^2^	80 (± 21)	66 (± 14)	75 (± 15)	0.07	1.00
RV-ESV index, ml/m^2^	39 (± 16)	24 (± 10)	28 (± 9)	**0.004**	**0.029**
LGE presence, *N* (%)	20 (100)	18 (90)	0 (0)	0.487	** < 0.001**
LGE extent, %	47 (± 34)	15 (± 11)	0 (± 0)	**0.002**	** < 0.001**

Bold indicates *p* < 0.05

### MyoTT findings compared to strain and mapping findings

**Fig. 2:**
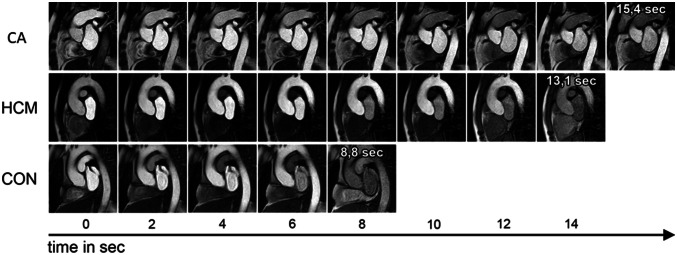
Schematic illustration of myocardial transit-time (MyoTT) depicting the different transit-time ‘through’ the myocardial microvasculature between cardiac amyloidosis (CA) patients (first row), hypertrophic cardiomyopathy (HCM) patients (second row), and healthy controls (third row)

MyoTT was significantly prolonged in patients with CA compared to both groups: 14.8 ± 4.1 s in CA vs. 12.2 ± 2.5 s in HCM (*p =* 0.043) vs. 7.2 ± 2.6 s in controls (*p* < 0.001), as illustrated in Fig. 2. Similar results were found for native T1 mapping and ECV measurement: both were significantly higher in CA (both *p* < 0.001). In addition, LV-GLS was also significantly impaired in CA compared to HCM as well as controls (Table 3).

**Table 3:** Myocardial transit-time (MyoTT) and novel CMR parameters

	CA patients	HNCM patients	Control group	*p* value (CA vs. HNCM)	*p* value (CA vs. control)
	*N =* 20	*N =* 20	*N =* 20		
Absolute MyoTT, sec	14.8 ± 4.1	12.2 ± 2.5	7.2 ± 2.6	**0.043**	** < 0.001**
MyoTT indexed to heart rate	0.214 ± 0.120	0.168 ± 0.050	0.085 ± 0.033	0.26	** < 0.001**
LV-GLS (3D), %	− 7.4 ± 3.8	− 10.2 ± 3.2	− 14.9 ± 2.0	**0.020**	** < 0.001**
Native T1 mapping, ms	1128 ± 86	1016 ± 41	996 ± 27	** < 0.001**	** < 0.001**
ECV, %	48 ± 12	30 ± 7	22 ± 5	** < 0.001**	** < 0.001**

Bold indicates *p* < 0.05

**Fig. 3:**
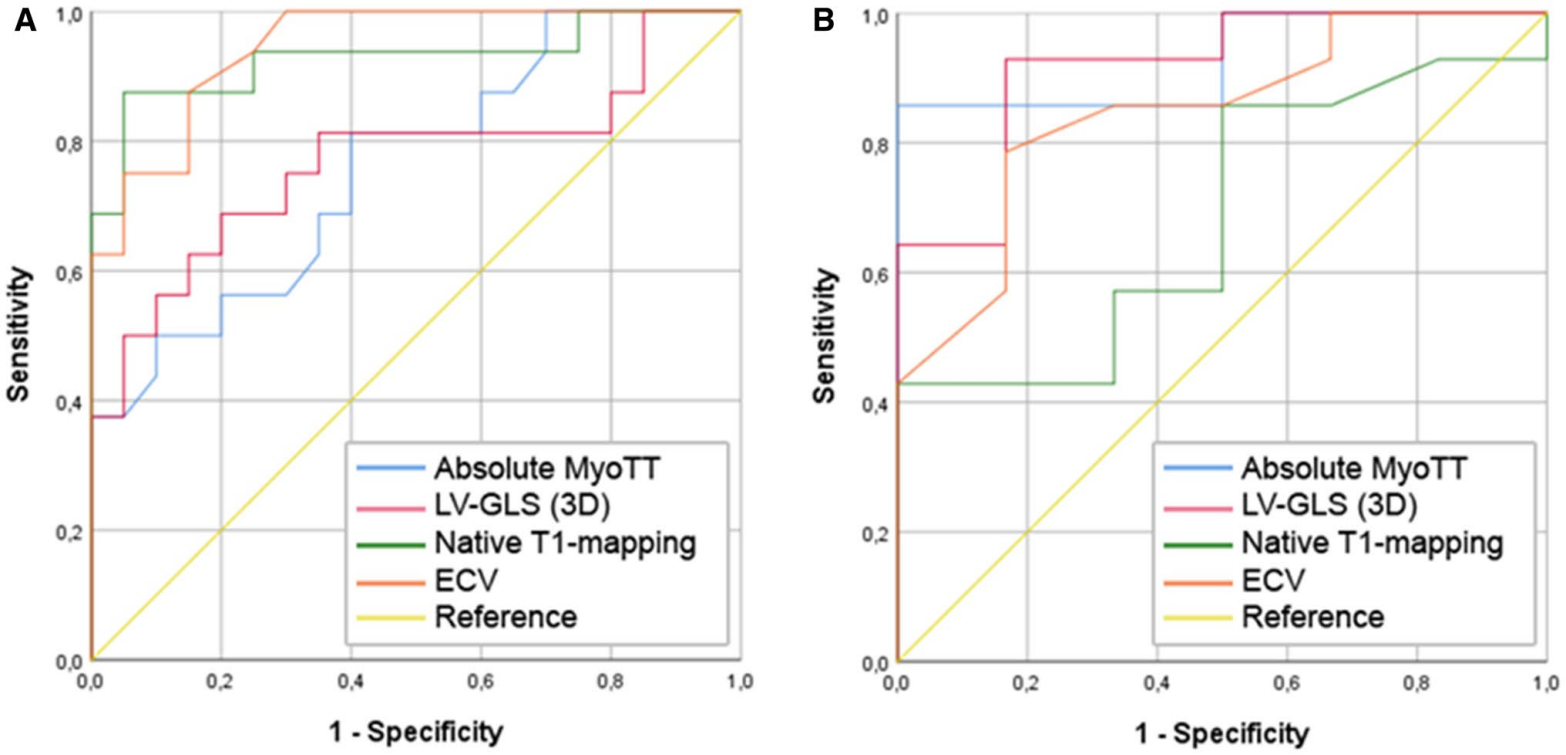
Receiver-operating characteristic (ROC) curves illustrating the diagnostic yield of myocardial transit-time (MyoTT) in comparison to left-ventricular global longitudinal strain (LV-GLS), native T1 mapping, and extracellular volume fraction (ECV) regarding the identification of cardiac amyloidosis (whole cohort, **a**) and hypertrophic cardiomyopathy (HCM and healthy controls, **b**)

In patients with CA, a significant—but not very strong—correlation was found between MyoTT and the extent of LGE (*r* = 0.490, *p =* 0.033), LV-GLS (*r* = 0.496, *p =* 0.031), and global ECV (*r* = 0.541, *p =* 0.030) (Table 4). Receiver-operating-characteristic (ROC) analysis was performed for all novel CMR parameters including MyoTT, LV-GLS, native T1 mapping, and global ECV regarding the delineation of CA (Fig. 3a,  Table 5): Global ECV showed the highest diagnostic yield with an area-under-the-curve (AUC) of 0.95 (*p* < 0.001). In contrast, MyoTT showed a higher diagnostic yield than all the other CMR parameters (including global ECV) regarding the differentiation of patients with “conventional” HCM from healthy controls with an AUC of 0.93 (*p =* 0.003) (Fig. 3b).

**Table 4:** Correlation between myocardial transit-time (MyoTT) and other clinical and CMR parameters in patients with cardiac amyloidosis (CA)

	CA patients (*N =* 20)	
	*r*	*p* value
Clinical parameters		
Male, *N* (%)	− 0.051	0.83
Age, years	0.401	0.08
Hypertension, *N* (%)	0.468	**0.037**
Diabetes, *N* (%)	0.064	0.79
High cholesterol, *N* (%)	0.303	0.19
Current smoker, *N* (%)	− 0.345	0.14
Conventional CMR parameters		
LV-EF, %	− 0.189	0.42
LV-EDV index, ml/m^2^	0.214	0.37
LV-ESV index, ml/m^2^	0.363	0.12
LV mass index, g/m^2^	0.541	**0.014**
Max. LV wall thickness, mm	0.453	**0.045**
RV-EF, %	− 0.093	0.70
RV-EDV index, ml/m^2^	0.290	0.21
RV-ESV index, ml/m^2^	0.294	0.21
LGE extent, %	0.490	**0.033**
Novel CMR parameters		
LV-GLS (3D), %	0.496	**0.031**
Native T1 mapping, ms	0.235	0.36
ECV, %	0.541	**0.030**

Bold indicates *p* < 0.05

**Table 5:** Delineation of hypertrophic cardiomyopathy (HCM) from controls as well as cardiac amyloidosis (CA)

	HNCM vs. control				HNCM vs CA			
	Cut-off	Sensitivity (%)	Specificity (%)	Accuracy (%)	Cut-off	Sensitivity (%)	Specificity (%)	Accuracy (%)
Absolute MyoTT, ms	9.1	86	66	85	14.7	50	86	65
Native T1 mapping, ms	1012	57	66	65	1051	88	79	82
ECV, %	25	79	83	80	33	88	79	83

Specifically, an MyoTT cut-off value of 9.1 s had an 86% sensitivity and 66% specificity (diagnostic accuracy 85%) in distinguishing HCM from controls (Table 5). In comparison, a cut-off value for native T1 of 1012 ms had a 57% sensitivity and 67% specificity (accuracy 65%), whereas an ECV value of 25% showed a 79% sensitivity and an 83% specificity in distinguishing HCM patients from controls (accuracy 80%). In contrast, regarding the differentiation of CA from HCM patients, an MyoTT cut-off value of 14.7 s showed a 50% sensitivity and 86% specificity (accuracy 65%), whereas a native T1 value of 1051 ms showed a 88% sensitivity and 79% specificity (accuracy 82%). The latter numbers are similar for ECV mapping and a cut-off value of 33%.

## Discussion

To the best of our knowledge, this is the first study that assesses the diagnostic yield of the novel CMR parameter MyoTT in both patients with CA and “conventional” HCM—in comparison to other established CMR parameters. The present study findings clearly show that mapping-based ECV is superior to MyoTT regarding the diagnosis of an “infiltrative” and predominantly “extracellular” disease such as CA. However, the present data also show that MyoTT is a very sensitive novel CMR parameter that allows both detection and characterization of cardiac diseases like “conventional” HCM that are characterized by less pronounced “extracellular” remodeling, but are rather dominated by “intracellular” and subsequent “intravascular” changes. In this context, MyoTT may not only help in the appropriate diagnosis and quantification of the severity of intra-/microvascular changes, but also be used to elegantly and easily monitor the course of the respective cardiac disease.

The assessment of CMD in non-ischemic cardiomyopathies has potential clinical value in different cardiomyopathies [30]. Briefly, the extent and severity of CMD correlates with worsening heart failure and poor clinical outcomes [31, 32]. In particular, hypertrophic heart diseases are characterized by CMD, and both “conventional” HCM [33] and cardiac amyloidosis [34] are typical examples. Obviously, there is a continuous interplay between myocardial perfusion abnormalities (particularly in the microvasculature) and myocardial tissue structure/remodeling. Novel CMR-methods such as T1 mapping and ECV measurement offer a unique non-invasive tool for myocardial tissue characterization and represent increasingly attractive techniques for clinicians in the work-up of cardiomyopathies. Changes in native T1-time and ECV value allow the assessment and quantification of primarily extracellular changes in the human myocardium [35]. In this context, different native T1- and ECV values were measured for the “conventional” HCM [36] and CA [37] patients. However, mapping and ECV measurements neither allow to directly assess “intravascular” changes nor to assess the degree of CMD. In contrast, MyoTT is a novel CMR parameter that promises to easily assess “intravascular” changes and quantify the degree of CMD.

In the case of an infiltrative cardiomyopathy like CA, structural changes of the extracellular space (in particular enlargement of ECV due to amyloid deposition) are expected to precede potential intravascular changes, resulting in CMD [34]. In addition, not only external compression of the coronary microvasculature by extracellular amyloid deposits but also additional amyloid deposits in the coronary vessel wall can increase the degree of CMD and induce severe myocardial ischemia even in the absence of epicardial obstructive CAD [38]. Therefore, the finding that the highest MyoTT values (reflecting severe CMD) were obtained in CA patients is not surprising. However, in the present study, native T1 mapping and ECV showed a higher diagnostic yield in comparison to MyoTT regarding the identification of patients with CA. This finding nicely illustrates that mapping and ECV measurement represent ideal imaging tools for the detection of cardiac diseases that are characterized by a predominant extracellular enlargement and support the aforementioned explanation that CMD (assessed by MyoTT) represents a less pronounced result of this extracellular process.

In contrast to CA, the histological characteristics of “conventional” HCM comprise myocyte hypertrophy and disarray as well as diffuse interstitial and perivascular fibrosis [39]. Coronary resistance increases in HCM not only due to “intracellular” enlargement with subsequent decrease in “extracellular” space but also due to vessel wall hypertrophy with subsequent reduction in luminal area, resulting in myocardial ischemia, myocyte death, and replacement fibrosis [17]. Noteworthy, the extracellular changes that are found in patients with advanced HCM are at least in part the result of abnormalities in the coronary microvasculature. Hence, the present finding that MyoTT is a sensitive parameter to differentiate patients with the conventional HCM from controls is not surprising and is backed up by the knowledge that the relative severity of CMD in relation to extracellular changes is more pronounced in HCM compared to CA. However, it must be emphasized that native T1 and ECV mapping are more useful techniques for the work-up of LV hypertrophy of unknown origin than MyoTT and provide a more reliable diagnosis of cardiac amyloidosis than MyoTT per se.

As shown in our previous study [28] and confirmed in the present one, the severity of CMD correlates with longitudinal strain in both HCM and CA. These findings are in line with the previous data that suggest an association between LV deformation behavior and myocardial ischemia in HCM patients [40]. Accordingly, the presence of both impaired longitudinal strain and CMD is frequently observed in CA patients, since an impaired longitudinal strain is partially due to disturbed microvascular function: the majority of longitudinal fibers are located in the subendocardium that in turn is more susceptible to (microvascular) ischemia [34]. Hence, the finding of a substantial correlation between MyoTT to longitudinal strain is of great interest: since abnormal myocardial deformation was shown to be associated with a higher rate of adverse cardiac events in HCM patients [41] and a worse survival in CA patients [42], a similar prognostic value can be deduced for the novel CMR parameter MyoTT. However, future studies are needed to prove this hypothesis.

## Limitations

It should be emphasized that a direct comparison regarding the diagnostic yield of MyoTT vs. routine CMR work-up (comprising cine- and LGE-imaging) for CA and/or HCM was not performed. Since the diagnostic value of the aforementioned routine CMR sequences in the assessment of unclear LV hypertrophy is already high, it is fair to assume that the addition of MyoTT will add only little to overall diagnostic certainty.

CA comprises different subtypes dependent on the precursor protein. For the purpose of this hypothesis-generating pilot study, no distinction was made between the different types of CA (AL and ATTR), because the underlying mechanism of cardiac involvement seems to be similar [43]. In addition, since CA is a rare disease, the size of our study group is limited [44]. Patients with obstructive CAD were excluded to minimize the risk of affecting measurement of MyoTT. Finally, the practical limitations of our novel MyoTT approach were already described in detail in our previous study [28].

## Conclusion

MyoTT provides additive information to the data that can be obtained from parametric mapping and strain measurement. The relative severity of CMD (measured by MyoTT) in relationship to extracellular changes (measured by ECV) is more pronounced in HCM compared to CA—in spite of a higher absolute MyoTT value in CA patients. Hence, MyoTT may improve our understanding of the interplay between extracellular/intracellular and intravasal changes that occur in the myocardium during the disease course of different cardiomyopathies.

## Data Availability

The data sets used and/or analyzed during the current study are available from the corresponding author on reasonable request.

## Abbreviations

AL: Immunoglobulin light chain amyloidosis
ATTR: Transthyretin amyloidosis
CA: Cardiac amyloidosis
CAD: Coronary artery disease
CMD: Coronary microvascular dysfunction
CMR: Cardiovascular magnetic resonance
CS: Coronary sinus
HCM: Hypertrophic cardiomyopathy
HF: Heart failure
IQR: Interquartile range
LGE: Late-gadolinium-enhancement
LV: Left ventricle
LV-EDV: Left-ventricular end-diastolic volume
LV-EF: Left-ventricular ejection fraction
MBF: Myocardial blood flow
MPR: Myocardial perfusion reserve
MyoTT: Myocardial transit-time
ROI: Region of interest
VENC: Velocity encoding
